# Evidence to guide the optimal timing for pre‐chemotherapy blood tests for early breast, colorectal cancer and diffuse large B‐cell lymphoma

**DOI:** 10.1002/cam4.4316

**Published:** 2021-09-28

**Authors:** Pinkie Chambers, Li Wei, Martin D. Forster, Emma Kipps, Ian C. K. Wong, Yogini Jani

**Affiliations:** ^1^ UCL School of Pharmacy University College London Hospitals NHS Foundation Trust London UK; ^2^ UCLH‐UCL Centre for Medicines Optimisation Research and Education London UK; ^3^ Department of Oncology UCL Cancer Institute University College London Hospitals NHS Foundation Trust London UK; ^4^ Royal Marsden Hospital London UK

**Keywords:** chemotherapy, monitoring, platelets, neutrophils, treatment‐delay

## Abstract

**Background:**

Re‐designing services and processes to meet growing demands in chemotherapy services is necessary with increasing treatments. There is little evidence guiding the timing and thresholds to be attained of pre‐chemotherapy blood assessments, namely neutrophils.

**Methods:**

A survey was developed and distributed to health professionals in the United Kingdom (UK) to examine current practice in timing and threshold values of neutrophils and platelets before treatment administration. This was followed by a retrospective cohort study, using data from electronic patient record systems; including patients initiating treatment between January 2013 and December 2018, to determine a safe timeframe for blood assessments; comparing neutrophil, platelet, creatinine and bilirubin levels at different time points.

**Results:**

The survey captured 25% of hospitals in the UK and variations were apparent in both the timing of assessments and thresholds needed, particularly for neutrophils. 616 (6.5%) of 4007 patients included had neutrophil levels measured twice within 7 days of treatment (with the first level taken beyond 3 days and the second test being within 3 days of treatment‐ the UK standard). Of the patients that attained an acceptable neutrophil level at their first test, five of the 616 (0.8%) became ineligible for administration from the test 2 level. 23% of patients improved their grade and became eligible for treatment. Little difference was observed for platelets.

**Conclusions:**

We have demonstrated that extending the timeframe for blood tests can be safe, however, this practice may cause unnecessary delays for patients if only an early test is relied on for eligibility.

## Graphical abstract

There is a dearth of evidence supporting the correct timing of blood tests prior to chemotherapy, leading to patients receiving numerous unnecessary tests. We aimed to provide this evidence, utilising data from electronic prescribing systems and showed that extending periods were safe but in the case of neutrophil assessments could lead to unwarranted delays.

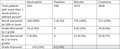

## 1 BACKGROUND

There is an immediate need to change current processes of care as chemotherapy patient numbers increase year upon year, posing demands on services. It is estimated that between 2018 and 2040, numbers of patients requiring first‐course chemotherapy, annually, will increase from 9.8 million to 15 million.^1^ To meet this rising demand, it is necessary to improve the way in which preparation and administration of treatment occur. An area that is commonly overlooked when making efficiencies to the service is the sparsity of evidence around the timing and threshold values of pre‐treatment blood tests.

Blood tests are required during any chemotherapy to ensure safety: ensuring adequate bone marrow, renal and hepatic function throughout chemotherapy.^2^, ^3^ Blood monitoring, particularly in the case of neutrophils, should be timed to be as close to the administration day as possible; however, this is not always practical. Wider timeframes for blood assessments would allow for more flexibility in treatment preparation and administration but there is uncertainty whether this would provide accurate results due to the occurrence of daily fluctuations in neutrophils for chemotherapy patients.^4^ Moreover, threshold values for tests such as pre‐treatment neutrophils have been reported to vary between clinicians,^5^, ^6^ where some clinicians choose higher threshold targets than others. This practice can mean disparities in the relative dose intensity of treatment received, which for some cancers can worsen a patients’ treatment outcome.^7^, ^8^

Many clinical trials have stipulated that it is acceptable for neutrophils to be assessed 48–72 hours^9^ prior to administration and so this has been generally regarded as an acceptable standard in the United Kingdom (UK). Likewise, a period of seven days is considered appropriate to assess the renal and liver functions. Anecdotally, some believe that the window of timing could safely be extended further, and any extension would be advantageous, allowing for advance planning. Evaluations undertaken by hospitals have shown that monitoring for neutrophils can be undertaken up to five days in advance of treatment without compromising safety.^10^ However, these evaluations have used small number of patients. Furthermore, without consensus in threshold values, extended timeframes may result in unnecessary treatment delays.

In the UK, it is common that a patient will have their blood assessed on a different day, but close to treatment, to streamline the prescribing and reconstitution of treatment.^11^ This strategy has been shown to reduce patient waiting times on the day of treatment.^12^ However, these blood tests can be scheduled on a day that coincides with a routine clinic visit that may be outside 72 h. When this occurs, another test will be taken just prior to administration and this practice allows us to retrospectively evaluate the timing of blood assessments. Electronic prescribing (EP) systems contain data to build evidence in these areas. Our aim was to use these data to understand whether the time window for assessing pre‐chemotherapy blood level could be extended without compromising patient safety. To support any findings, we also aimed to examine any variations between UK hospitals around threshold values for pre‐treatment neutrophils and platelets.

## 2 METHODS

To meet our aims, we conducted a retrospective descriptive study, using data from EP systems and a short survey intended to capture details of hospital policies in UK chemotherapy treating hospitals. We focussed both studies on specific common treatments used first line, in three cancers—early breast cancer, diffuse large b‐cell lymphoma and colorectal cancer. These were chosen firstly, as they have a high incidence in the UK^13^ and secondly as there is evidence that relative dose‐intensity is an indicator for improved outcomes in these cancers. A dataset was collected and formed part of a wider study to develop a risk prediction model to determine patients likely to encounter dose delays, as a future objective. The survey was designed to be online, short in length and very specific to the treatments specified in the inclusion criteria for the data study. As the aim of the study was to capture hospital policies, we included a question to capture the hospital name. Distribution was via email and snowball sampling through two professional societies—The Association of Cancer Physicians and The British Oncology Pharmacists Association. The survey was open for a one‐month period 1 July 2020 to 31 July 2020 and a reminder email was sent through the societies after two weeks of the first to increase participation.

### 2.1 Inclusion and exclusion criteria

#### 2.1.1 Survey

Health care professionals working in the chemotherapy services, including medical and clinical oncologists (registrar or consultant level), haematologists (registrar or consultant level) chemotherapy nurses, clinical nurse specialist, oncology and haematology pharmacists. Participants needed to respond in English. We allowed multiple professionals from the same hospital to participate in the survey and intended to use this data to understand any inter‐hospital variation in practice.

#### 2.1.2 Data study

Three large, geographically diverse, hospitals in England provided data for this study. Patients’ records were included if they were aged 18 or over. Patients were identified through the chemotherapy EP system at each site and all data were extracted for the period January 2013 to December 2018. We used the first‐chemotherapy treatment from the EP data as the index date for entry to the cohort during the study period and patients were followed up until the administration of the sixth cycle of treatment.

Data were restricted to the following three tumour groups: breast, colorectal and diffuse large B‐Cell lymphoma, identified using the ICD10 codes C50, C83, C19, C19, C20 and C21. Patients were only included if they received first‐line treatment with the following regimens: epirubicin and cyclophosphamide (EC) only or with fluorouracil (FEC); docetaxel alone (as sometime given for three cycles before EC or FEC), these were chosen as they were standard of care first‐line therapies in the UK during the collection period; irinotecan modified de Gramont (IRMDG); oxaliplatin modified de Gramont (FOLFOX); oxaliplatin and capecitabine (OXCAP); and rituximab, cyclophosphamide, vincristine and prednisolone (RCHOP).

Patients were excluded if they received only one cycle of treatment. These were excluded by researcher PC following data transfer. Additionally, we excluded patients where the second cycle was administered beyond a period of 60 days from the date of first treatment as this type of delay was outside the scope of this research.

### 2.2 Analysis

#### 2.2.1 Survey

Data were extracted from Qualtrics®, the online survey platform and analysed using STATA 15. Results are presented as counts and percentages (%). As more than one respondent could answer from a hospital, answers from multiple respondents from the same hospital were grouped and agreement was calculated where warranted. Answers were grouped according to the time interval between blood tests and treatment: less than and including 2 days, 3 days and 4–7 days. Similarly, for threshold values, a value of 1 × 10^9^/L and 100 × 10^9^/L as index values for neutrophils and platelets respectively were used and groups created to be below or greater than this index value, retaining separate groups under, the same as and greater than the index. These values were used as they were the lowest standard threshold values for treatments reported by the three hospitals recruited to the data study. Those that answered that a policy around timings or threshold was not available at their hospital or the regimen was not used, were grouped separately.

#### 2.2.2 Data study

We compared changes in blood count values using The Common Terminology Criteria for Adverse Events (CTCAE) grading.^14^ We considered day 1 (the date of the first cycle) as the index date and each blood‐test date was ordered in terms of days from the index. Baseline results were any results that either preceded the index date by up to 7 days or were taken within 72 h following the index. In the event that there was more than one baseline value available, we used the closest value to the index.

Regimens included were categorised by cycle length, a standard interval for a particular regimen. By using the standard cycle length of either 14 or 21 days, we were able to determine if treatment administration had been delayed. We only used data for two administrations (cycles) as the blood testing data for these were the most complete. Additionally, it is understood that the most profound changes in neutrophil values occur following the first administration.^15^ However, we were able to report administrations and timings of subsequent cycles in our analysis, in addition to any dose alterations. Patient outcome data (progression, death) was not collected.

Test results used in the analysis were neutrophil count, bilirubin and creatinine levels, with the latter two as measures of liver and renal function respectively.

##### 2.2.2.1 Neutrophils and platelets

We grouped the days of the blood tests for neutrophils and platelets. For patients on a 21‐day treatment cycle, we considered a blood result from days 15 to 18 as being outside the approved period (Test 1). A blood result from days 19 to 22 was within the approved period (Test 2). For patients on a 14‐day cycle, a blood test within 2 days of treatment is considered standard of care, therefore a blood result from days 11 or 12 were outside the approved period (Test 1), while a blood result from days 13 to 15 were within the approved period (Test 2). If there were two tests within the same grouping, we chose the closest value to the treatment date. Each neutrophil value was categorised as per CTCAE grading.

Additionally, for all the regimens, we included the absolute neutrophil count (ANC) threshold of 1 × 10^9^/L, and platelets 100 × 10^9^/L, to determine whether treatment would be administered or not. We used this threshold to understand if test 1 would have resulted in the same treatment decision as test 2 using Fisher's exact test. In the scenarios where the earlier test would result in treatment being given but the results of test 2 would have differed, we described the impact to the patients’ subsequent treatments.

##### 2.2.2.2 Creatinine and bilirubin

We investigated the changes in creatinine and bilirubin from baseline results to just prior administration of the second cycle of treatment, to detect any significant grade change, defined using CTCAE grade changes, warranting amendments or reductions. For creatinine in particular, in practice, a clinician may commonly choose to monitor patients more intensely when a 10% or more change is noted and therefore, we reported numbers for this.

### 2.3 Missing data

We only analysed patients that had complete data needed for our analysis for both the data and survey study.

## 3 RESULTS

### 3.1 Survey

Two hundred eight participants opened the survey, however, of these only 91 completed it. Seventy‐seven of the 91 participants belonged to separate public‐funded hospitals in England, Scotland, Wales and Northern Ireland, which accounted for approximately 25% of the hospitals that deliver chemotherapy in these countries. Table S1 shows the breakdown of professional groups that started and completed the survey. There were five hospitals where more than one health professional completed the survey, where this was the case, these HCPs were reporting for different tumour types and therefore inter‐hospital variability could not be assessed.

**FIGURE 1:**
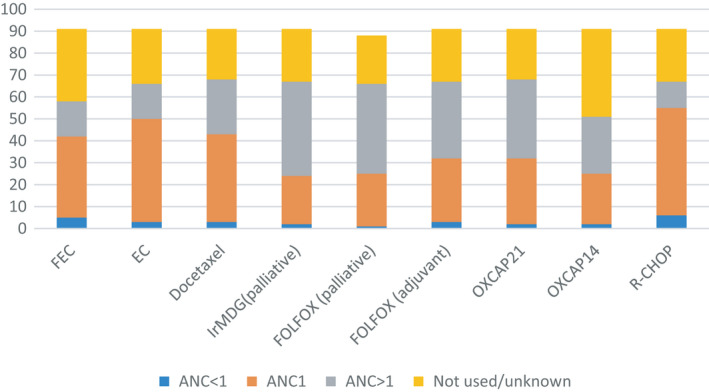
Number of respondents using threshold values below 1, of 1 and greater than 1 for different chemotherapy treatments. FEC, fluorouracil, epirubicin and cyclophosphamide; T FEC, docetaxal, fluorouracil, epirubicin and cyclophosphamide; R‐CHOP, rituximab, cyclophosphamide, doxorubicin, vincristine and prednisolone; FOLFOXIRI, fluorouracil, oxaliplatin and irinotecan; IRMDG, irinotecan and fluorouracil; OXCAP, oxaliplatin and capecitabine where 14 and 21 refer to the respective cycle

Table 1 displays the differences in policies relating to the timing of blood tests to assess a neutrophil and platelet values prior to treatment. In the majority of participating hospitals, the standard of care was for this test to be taken within 3 days of treatment for breast cancer; however, 15% of HCPs reported requiring a threshold of greater than 1 × 10^9^/L (Figure 1). Interestingly, for the treatments associated with 14‐days cycles in the colorectal cancers, many hospitals opted for a closer test (1–2 days pre‐treatment (27–29%) whereas others had a standard for over 4‐days (15–19%). Moreover, a greater proportion of HCPs reported standard thresholds of >1 × 10^9^/L compared to 1 × 10^9^/L in both the palliative and adjuvant settings.

**TABLE 1:** Number of days pre‐treatment a patient would have a blood test

Chemotherapy	Within 1–2 days	3 days	4 days or above	No guidance	Not used/unknown
FEC *N* = 91	20 (22%)	25 (27%)	13 (14%)	4 (4%)	29 (32%)
EC *N* = 91	23 (25%)	29 (32%)	14 (15%)	3 (3%)	22 (24%)
Docetaxel *N* = 91	23 (25%)	31 (34%)	15 (16%)	3 (3%)	19 (21%)
IrMDG (palliative) *N* = 91	26 (29%)	26 (29%)	15 (16%)	2 (2%)	22 (24%)
FOLFOX (Palliative) *N* = 91	26 (29%)	26 (29%)	15 (16%)	2 (2%)	22 (24%)
FOLFOX^a^ (Adjuvant) *N* = 88	25 (27%)	27 (30%)	14 (15%)	1 (1%)	21 (24%)
OXCAP21 *N* = 91	24 (26%)	27 (30%)	17 (19%)	2 (2%)	21 (23%)
OXCAP14 *N* = 91	22 (24%)	22 (24%)	13 (14%)	1 (1%)	33 (36%)
R‐CHOP *N* = 91	13 (14%)	22 (24%)	29 (32%)	2 (2%)	25 (27%)

Abbreviations: FEC, fluorouracil, epirubicin and cyclophosphamide; FOLFOX, oxaliplatin and fluorouracil for palliative and adjuvant indications; FOLFOXIRI, fluorouracil, oxaliplatin and irinotecan; IRMDG, irinotecan and fluorouracil; OXCAP, oxaliplatin and capecitabine, where 14 and 21 refer to the respective cycle length; R‐CHOP, rituximab, cyclophosphamide, doxorubicin, vincristine and prednisolone; T FEC, docetaxal, fluorouracil, epirubicin and cyclophosphamide.

^a^ Three respondents missed this question.

Around a third of HCPs reporting on timeframes regimen RCHOP, used for diffuse large b call lymphomas, reported that 4 days or above was their standard timing between blood test and treatment but 15% required a threshold above 1 × 10^9^/L for neutrophils to commence treatment. Threshold values for platelets are displayed in supplementary Figure 1.

34% (*N* = 31) HCPs commented at the end of the survey; 26 relating to patient centred exceptions to policies that would be the decision of the prescriber, meaning that where thresholds were not met the prescriber could be contacted to decide whether treatment should proceed.

### 3.2 Data study

A total of 4007 patients were included in the analysis from three hospitals, of which 66% were female, consistent with a large proportion of patients having breast cancer (40%). Forty‐five per cent of the patients were receiving treatment for colorectal cancer. In total 1291 (32%) of patients received a colony stimulating factor (CSF) at their first treatment cycle, a breakdown by treatment regimen is available in Table S2. 6.5% of patients had two neutrophil results within 7 days of treatment administration but the repeated test may have been a consequence of treatment hospital policy. Table 2 gives an overview of patients.

**TABLE 2:** Overview of patient characteristics from data study

Parameter	Patients without two neutrophil results	Patients with two neutrophil counts
Number of patients (*N*)	3391	616
Hospital	Hospital 1: 1690 (50%)	Hospital 1: 75 (12%)
	Hospital 2: 1178 (34%)	Hospital 2: 107 (17%)
	Hospital 3: 523 (15%)	Hospital 3: 434 (70%)
Age	Median 56 (18–90)	56 (18–90)
Gender	Female: 2242 (66%)	395 (64%)
	Male: 1149 (34%)	221 (36%)
Tumour type	Breast: 1441 (42%)	Breast: 1618 (40%)
	DLBCL: 363 (11%)	DLBCL: 572 (14%)
	Colorectal: 1587 (47%)	Colorectal: 1817 (45%)
Regimen received	FEC: 713 (21%)	FEC: 130 (21%)
	EC: 501 (15%)	EC: 17 (3%)
	T‐FEC: 230 (7%)	T‐FEC: 31 (5%)
	^a^RCHOP: 363 (11%)	^a^RCHOP: 207 (33%)
	FOLFOXIRI: 8 (0.2%)	FOLFOXIRI: 13 (2%)
	IRMDG: 576 (17%)	IRMDG: 631 (9%)
	^a^OXCAP: 324 (10%)	^a^OXCAP: 32 (5%)
	OXMDG: 679 (20%)	OXMDG: 809 (22%)
Patients receiving cycle 1 treatment delay	539 (20%)	
Baseline absolute neutrophil count	Median 4.62 range (0.4–72)	Median 4.63 range (0.5–51)
Performance status	0–2: −3384 (99.8%)	0–2: 611 (99%)
	>2: 7 (0.2%)	>2: 5 (0.8%)
Chemotherapy cycle length		
14 days	1355 (40%)	204 (33%)
21 days	2036 (60%)	412 (67%)

Abbreviations: DLBCL, diffuse large B cell lymphoma; FEC, fluorouracil, epirubicin and cyclophosphamide; T FEC, docetaxal, fluorouracil, epirubicin and cyclophosphamide; R‐CHOP, rituximab, cyclophosphamide, doxorubicin, vincristine and prednisolone; FOLFOXIRI, fluorouracil, oxaliplatin and irinotecan; IRMDG, irinotecan and fluorouracil; OXCAP, oxaliplatin and capecitabine; OXMDG, oxaliplatin and fluorouracil.

^a^ Combined for patients on 14 and 21 days schedule.

Table 3 shows that in 40% of patients’ neutrophils reduced by 10% or more; however, grade was only worsened in 2.6% of patients. The downward trend could signify delayed nadirs in some patients; however, Table 3 shows that relatively few patients experienced changes that would impact treatment administration. We conducted a Fisher's exact test and reported a *p*‐value of 0.62, demonstrating no statistical significance between the repeated tests taken at these different periods. However, there may still be clinical significance.

**TABLE 3:** Grade changes in neutrophils, bilirubin and creatinine values

	Neutrophils	Platelets	Bilirubin	Creatinine
Total patients with more than 1 result within a defined period^a^	616	436	3973	3828
Result worsened by 10% or more	246 (40%)	1 (0.2%)	725 (18%)	721 (19%)
Grade worsened by 1 grade	16 (2.6%)	0	6 (0.15%)	24 (0.6%)
Grade Worsened by 2 or more grades	5 (0.8%)	0	12 (0.3%)	25 (0.7%)
Grade improved	142 (23%)	6 (1.4%)		

^a^ Neutrophil grade changes between two levels taken within 7 days; both prior to the second chemotherapy administration. Creatinine and bilirubin change prior to first and second chemotherapy cycles. Grade improvements in creatinine and bilirubin are not applicable therefore not reported.

Five patients of the 616 that had two tests did not meet the threshold value to receive treatment on their second test, when their earlier test (taken beyond the 72‐h period) had indicated a value above the threshold (see Table 4). Three of the 5 patients fell marginally short of the threshold of 1 × 10^9^/L, but with neutrophils greater than 0.9 × 10^9^/L. These three patients had a record of receiving chemotherapy without delay or future delay. One further patient had a record of receiving treatment (EC) but subsequent cycles were not recorded. The final patient, receiving FEC, received a dose reduction of 25% at cycle 2 and no further cycles were recorded in their treatment record.

**TABLE 4:** Showing those eligible for treatment at test 1 and test 2

	Test 2: ANC ≥ 1 × 10^9^/L	Test 2: ANC < 1 × 10^9^/L
Test 1: ANC ≥ 1 × 10^9^/L	498 (81%)	5 (0.8%)
Test 1: ANC < 1 × 10^9^/L	111 (18%)	2 (0.3%)

Abbreviation: ANC, absolute neutrophil count.

A subgroup analysis of creatinine and bilirubin is presented in Table 5 and shows that in those patients with breast cancer, there were no cases of grade changes for bilirubin and very few changes for the other cancers studied.

**TABLE 5:** Renal and liver function difference in grade by tumour group

	Cancer	Total patients	1 grade change	2 or more grade change
Creatinine (*n* = 3973)	Breast	1618	4 (0.3%)	3 (0.2%)
	Colorectal	1817	11 (0.6%)	15 (0.8%)
	DLBCL	572	9 (1.6%)	7 (1.2%)
Bilirubin (*n* = 3828)	Breast	1618	0 (0%)	0 (0%)
	Colorectal	1796	5 (0.3%)	10 (0.28%)
	DLBCL	564	1 (0.2%)	2 (0.4%)

## 4 DISCUSSION

The purpose of this work was to guide practice changes around pre‐chemotherapy blood assessments, to ensure chemotherapy services can manage with increasing demands. The survey we undertook underlined an existing variation in policies nationally in the UK resulting from the lack of evidence and consensus in the area. Our data study assured us that extensions to time‐periods beyond the standard of 72 h to assess for myelosuppression are safe, however, there may be other consequences to this practice, including patients not attaining their threshold values and being unduly delayed from treatment or requiring additional testing closer to the treatment date.

We found that in patients who had two tests taken within 7 days of their planned treatment date, less than 1% of patients had a CTCAE Grade worsened by 2 or more. Although statistically there was no difference between a neutrophil count taken at an earlier period in 23% of cases blood tests taken closer to the treatment day showed grade improvements for neutropenia. An earlier assessment might have caused the dose to be wrongly withheld, meaning more patient visits. The survey showed the variation in threshold values and where some hospitals have a standard policy of 1 × 10^9^/L, a patients’ likelihood of an unnecessary delay increases. Relative dose intensity has been by many to be important to treatment outcomes and appropriate thresholds and consistency in timings will assure equity in treatments received.

Apart from conference proceedings,^10^, ^16^, ^17^ we found little evidence to support the practice of extending the time window of blood tests beyond 72 h. One study from the United States investigated blood assessments prior to initiation of chemotherapy and concluded that a 7‐day window was safe^18^ but did not investigate subsequent dosing. Another small study^19^ of 27 patients receiving bortezemib treatment investigated whether blood tests could be reduced in frequency. Authors here concluded that a reduced frequency could be achieved when values for neutrophils and platelets were at an adequate level on treatment initiation. Our study is comparable with others; however, our large sample allowed us to highlight potential clinical implications in extending time periods. Drops beyond threshold values are very rare and may not be captured in small single‐site evaluations.

Our work is the first of its kind in presenting national variations in practice and a descriptive analysis of hospital data that can be a goad to developing national consensus. There are however limitations to our findings. We distributed the survey through two national societies, where membership is optional, and members may not be representative of the national workforce. As reported a large proportion of participants failed to complete the survey and this may have been as they did not want to disclose where they worked or they did not have any policies in place in this area. We were limited by the number of patients who had these repeated blood results available on EP systems. Demographic differences between those that had repeated assessments and those that had were not seen, but there were differences between hospitals, which could relate to individual hospital policy for example the use of colony‐stimulating factors. A further limitation was that without access to medical notes we were unclear as to any admissions or serious adverse event that may have subsequently occurred; we could only report impact on subsequent treatments. The EP system data used provided us with detailed information on blood assessments but as patients can move between hospitals for subsequent lines of treatment and death is not accurately recorded we were unable to accurately collect and report this data.

All hospitals are challenged with adapting policies and patient flows to protect patients even where blood assessments are routinely taken on the day of treatment. Our results can help hospitals make informed decisions in developing new patient pathways to minimise interactions and patient waits. Implementation of our results will be dependent on the individual hospital facilities such as the availability of point‐of‐care blood testing and the time taken to obtain results for tests. Benefits of reducing the frequency of some kidney and liver function tests include reduced cost and reduced interventions for the patient; the cost implications are an area of future research.

## 5 CONCLUSIONS

Variation in the UK exists in both the threshold values and in the timing of pre‐treatment assessments for neutrophils and this could cause regional disparities in treatment intensity. Routine pre‐chemotherapy blood tests should be undertaken within 2–3 days of treatment (dependant on cycle length) to avoid multiple patient visits to hospitals and unwarranted delays. For many patients studied, the assessment of renal and liver function at every cycle was unjustified.

## ETHICS AND DATA USE

The data study was based on retrospective datasets; Health Research Authority (HRA) approvals were required and granted on 24 November 2017 (IRAS 226078). Information governance approvals were granted at each recruited site in accordance with hospital policies.

## CONFLICTS OF INTEREST

Authors Li Wei, Yogini Jani and Emma Kipps report no conflicts of interest; Martin D Forster reports grants and honoraria from, AstraZeneca, Bristol Myers Squibb, Celgene, Eli‐Lilly, Merck, MSD, Nanobiotix, Novartis, Pfizer, Roche and Takeda; outside the submitted work; Pinkie Chambers reports research grants from Janssen, Pfizer, Tessaro and Bristol Myers Squibb; outside the submitted work. Professor Wong reports research grants from Janssen and Bristol Myers Squibb; outside the submitted work.

## Supporting information

Supplementary Material

## Data Availability

The datasets generated during and analysed during the current study are available from the corresponding author on reasonable request.

## References

[1] Wilson BE , Jacob S , Yap ML , Ferlay J , Bray F , Barton MB . Estimates of global chemotherapy demands and corresponding physician workforce requirements for 2018 and 2040: a population‐based study. Lancet Oncol. 2019;20(6):769‐780. 31078462 10.1016/S1470-2045(19)30163-9

[2] Duong CD , Loh JY . Laboratory monitoring in oncology. J Oncol Pharm Pract. 2006;12(4):223‐236. 17156594 10.1177/1078155206072982

[3] Krens SD , Lassche G , Jansman FGA , et al. Dose recommendations for anticancer drugs in patients with renal or hepatic impairment. Lancet Oncol. 2019;20(4):e200‐e207. 30942181 10.1016/S1470-2045(19)30145-7

[4] Dunwoodie EH . Home Testing of Blood Counts in Patients with Cancer. Ethesis University of Leeds; 2018. https://etheses.whiterose.ac.uk/22915/1/Dunwoodie_E_Medicine_MD_2018.pdf

[5] Kogan LG , Davis SL , Brooks GA . Treatment delays during FOLFOX chemotherapy in patients with colorectal cancer: a multicenter retrospective analysis. J Gastrointest Oncol. 2019;10(5):841‐846. 31602321 10.21037/jgo.2019.07.03 PMC6776798

[6] Chiarotto JA , Dranitsaris G . A community hospital‐based study: a prespecified neutrophil count with adjuvant mFOLFOX6 associated with increased delays, increased G‐CSF use, and reduced dose intensity. Cancer Manag Res. 2021;13:4087‐4094. 34040446 10.2147/CMAR.S307713 PMC8141389

[7] Citron ML . Dose‐dense chemotherapy: principles, clinical results and future perspectives. Breast Care (Basel). 2008;3(4):251‐255. 21076605 10.1159/000148914 PMC2974980

[8] Lyman GH . Impact of chemotherapy dose intensity on cancer patient outcomes. J Natl Compr Canc Netw. 2009;7(1):99‐108. 19176210 10.6004/jnccn.2009.0009

[9] National Institute of Healthcare Research Chemotherapy and Pharmacy Advisory Service . Guidance on the Drug Related Content of Clinical Trial Protocols 2015; 2015. https://www.nihr.ac.uk/nihr‐in‐your‐area/cancer/cpas.htm

[10] Thwaites BME , Chambers P , Ford N . Assessing the safety of an increase in validity of full blood counts from 72 to 96 h in our breast cancer chemotherapy patients. J Oncol Pharm Pract. 2017;23(8_suppl):1–70.

[11] Chambers PJS , Gallagher C . The Chemotherapy Services Audit; 2013. London Cancer. http://www.londoncancer.org/media/84650/london‐cancer‐chemotherapy‐audit‐2013‐july‐2014‐.pdf

[12] Kallen MA , Terrell JA , Lewis‐Patterson P , Hwang JP . Improving wait time for chemotherapy in an outpatient clinic at a comprehensive cancer center. J Oncol Pract. 2012;8(1):e1‐e7. 22548015 10.1200/JOP.2011.000281 PMC3266321

[13] Bray F , Ferlay J , Soerjomataram I , Siegel RL , Torre LA , Jemal A . Global cancer statistics 2018: GLOBOCAN estimates of incidence and mortality worldwide for 36 cancers in 185 countries. CA Cancer J Clin. 2018;68(6):394‐424. 30207593 10.3322/caac.21492

[14] National Cancer Institute . Common Terminology Criteria for Adverse Events (CTCAE) version 5 2017; 2017. https://ctep.cancer.gov/protocolDevelopment/electronic_applications/ctc.htm#ctc_50

[15] Lyman GH , Kuderer NM , Crawford J , et al. Predicting individual risk of neutropenic complications in patients receiving cancer chemotherapy. Cancer. 2011;117(9):1917‐1927. 21509769 10.1002/cncr.25691 PMC3640637

[16] Bayliss K . Abstract: pilot study: incidence of hyperbilirubinaemia insarcoma patients receiving single agent. J Oncol Pharm Pract. 2017;23(suppl 8):1‐70.

[17] Agapinaki EYL , Streetly M . Full blood count review prior to bortezomib dosing at Guy’s Hospital. J Oncol Pharm Pract. 2020;25(suppl 8):1‐116.

[18] Warr J , Hird AE , DeAngelis C , Giotis A , Ko YJ . Baseline blood work before initiation of chemotherapy: what is safe in the real world? J Oncol Pract. 2013;9(5):e182‐e185. 24045345 10.1200/JOP.2012.000719

[19] Waight CC , Cain R . Authorising bortezomib treatment prior to reviewing haematology results: a step toward home administration. J Oncol Pharm Pract. 2014;20(5):351‐355. 24154652 10.1177/1078155213508438

