# Supplementary material for: Evidence to guide the optimal timing for pre‐chemotherapy blood tests for early breast, colorectal cancer and diffuse large B‐cell lymphoma
Source: Cancer Med. 2021 Sep 28;10(22):7996–8004. doi: 10.1002/cam4.4316 (PMC8607255; doi:10.1002/cam4.4316)
Supplement: Supplementary file 1 — Supplementary Material [file CAM4-10-7996-s001.docx]

Supplementary Figure 1. Showing number of respondents using threshold values below 100 100 x 10^9^ /L, of 100 100 x 10^9^ /L and greater than 100 100 x 10^9^ /L for different chemotherapy treatments

Abbreviations:FEC- fluorouracil, epirubicin and cyclophosphamide; R-CHOP- rituximab, cyclophosphamide, doxorubicin, vincristine and prednisolone, FOLFOXIRI fluorouracil, oxaliplatin and irinotecan; IRMDG- irinotecan and fluorouracil; OXCAP- oxaliplatin and capecitabine where 14 and 21 refer to the respective cycle length; FOLFOX oxaliplatin and fluorouracil for palliative and adjuvant indications

Supplementary table 1. Professional groups completing the survey

| **Professional group** | **Number starting survey** | **Number completing survey** |
| --- | --- | --- |
| Pharmacist | 107 (63%) | 55 (60%) |
| Oncologist (medical or clinical) | 40 (23%) | 23 (25%) |
| Chemotherapy nurse or Clinical nurse specialist | 14 (8%) | 8 (9%) |
| Haematologist | 3 (2%) | 2 (2%) |
| Other (unspecified) | 6 (4%) | 3 (3%) |
| Total participants | 170 (100%) | 91 (100%) |

**Supplementary table 2. Colony Stimulating Factors received at cycle 1, by chemotherapy regimen**

| **Cancer** | **Treatment** | CSF administered | CSF not administered |
| --- | --- | --- | --- |
| Breast | EC | 447 (86%) | 73 (14%) |
|  | FEC | 574 (68%) | 266(32%) |
|  | Docetaxel | 162 (62%) | 96 (38%) |
| Colorectal | FOLFOXIRI | 2 (10%) | 19 (90%) |
|  | IRMDG | 11 (2%) | 620 (98%) |
|  | OXCAP | 2 (1%) | 354 (99%) |
|  | FOLFOX | 19 (2%) | 790 (98%) |
| DLBCL | RCHOP | 74(13%) | 498 (87%) |
| Total | | 1291 (32%) | 2716 (68%) |

Abbreviations: CSF – colony stimulating factor;FEC- fluorouracil, epirubicin and cyclophosphamide; R-CHOP- rituximab, cyclophosphamide, doxorubicin, vincristine and prednisolone, FOLFOXIRI fluorouracil, oxaliplatin and irinotecan; IRMDG- irinotecan and fluorouracil; OXCAP- oxaliplatin and capecitabine where 14 and 21 refer to the respective cycle length; FOLFOX oxaliplatin and fluorouracil for palliative and adjuvant indications
